# MMP-3 expression and release by rheumatoid arthritis fibroblast-like synoviocytes induced with a bacterial ligand of integrin α5β1

**DOI:** 10.1186/ar1462

**Published:** 2004-11-24

**Authors:** Mirjam B Zeisel, Vanessa A Druet, Dominique Wachsmann, Jean Sibilia

**Affiliations:** 1 Inserm 392, Infection et Inflammation, Université Louis Pasteur de Strasbourg, Faculté de Pharmacie, 74 route du Rhin, 67400 Illkirch, France; 2 Département de Rhumatologie, Hôpitaux Universitaires de Strasbourg, Strasbourg, France

**Keywords:** fibroblast-like synoviocytes, integrin α5β1, MMP-3, PAMP

## Abstract

Fibroblast-like synoviocytes (FLSs) play a major role in the pathogenesis of rheumatoid arthritis (RA) by secreting effector molecules that promote inflammation and joint destruction. How these cells become and remain activated is still elusive. Both genetic and environmental factors probably play a role in transforming FLSs into inflammatory matrix-degrading cells. As bacterial products have been detected in the joint and shown to trigger joint inflammation, this study was undertaken to investigate whether a bacterial ligand of integrin α5β1, protein I/II, could contribute to the aggressive behavior of RA FLSs. Protein I/II is a pathogen-associated molecular pattern (PAMP) isolated from oral streptococci that have been identified in the joints of RA patients. The response of RA and osteoarthritis FLSs to protein I/II was analyzed using human cancer cDNA expression arrays. RT-PCR and pro-MMP-3 (pro-matrix metalloproteinase) assays were then performed to confirm the up-regulation of gene expression. Protein I/II modulated about 6% of all profiled genes. Three of these, those encoding IL-6, leukemia inhibitory factor, and MMP-3, showed a high expression level in all RA FLSs tested, whereas the expression of genes encoding other members of the cytokine or MMP-family was not affected. Furthermore, the up-regulation of MMP-3 gene expression was followed by an increase of pro-MMP-3 release. The expression of interferon regulatory factor 1 and fibroblast growth factor-5 was also up-regulated, although the expression levels were lower. Only one gene, that for insulin-like growth factor binding protein-4, was down-regulated in all RA FLSs. In contrast, in osteoarthritis FLSs only one gene, that for IL-6, was modulated. These results suggest that a bacterial ligand of integrin α5β1 may contribute to the aggressive behavior of RA FLSs by inducing the release of pro-inflammatory cytokines and a cartilage-degrading enzyme, such as IL-6 and MMP-3, respectively.

## Introduction

Fibroblast-like synoviocytes (FLSs) appear to play a major role in the pathogenesis of rheumatoid arthritis (RA). These cells are characterized by pannus formation, cartilage invasion, and secretion of effector molecules, including cytokines and chemokines, that act on various cells to promote inflammation [1-3]. Recent experiments have shown that although FLSs were able to secrete large amounts of IL-6 and IL-8, they failed to release significant amounts of TNF-α, IL-1, IL-15, and IL-18 [4,5]. They showed a dissociated pattern of cytokine mRNA and protein expression, suggesting the existence of post-transcriptional regulation. FLSs are also the principal promoters of joint destruction [3], either through the release of proteolytic enzymes such as matrix metalloproteinases (MMPs), or indirectly through the stimulation of osteoclastogenesis. FLSs are the source of a broad range of MMPs, including MMP-1, MMP-13, and MMP-3; the last of these degrades different types of collagen and proteoglycans and activates other MMPs such as MMP-2 and MMP-9.

How these cells become and remain activated is still not known. Numerous factors may account for the permanent changes observed in FLS functions. To date it is very difficult to define whether FLSs are changed in response to genetic modifications, such as mutations or microsatellite instability, or in response to environmental factors. Both factors probably play a role in transforming FLSs into invading, inflammatory, matrix-degrading cells. Exposure to an inflammatory environment may even result in DNA damage [6]. On the other hand, genetic modifications may contribute to inflammation: it has been shown that a decrease of the expression of p21, an inhibitor of the cyclin-dependent kinases which is regulated by p53, activates activating protein-1, leading to enhanced cytokine and MMP synthesis in RA FLSs [7,8].

Regarding environmental factors, there has been considerable interest in a possible role of innate immunity in the initiation and perpetuation of inflammation during RA. Bacterial products – also called pathogen-associated molecular patterns (PAMPs) – such as lipopolysaccharide (LPS), peptidoglycan, and bacterial DNA have been detected in the joint [9,10] and were shown to trigger joint inflammation: in fact, oral administration of LPS exacerbates collagen-induced arthritis in mice, and intra-articular injection of CpG oligonucleotides and of peptidoglycan leads to transient arthritis in mice [11-13]. As DNA and rRNA from a wide variety of bacterial species have been identified in the joints of RA patients [9,14,15], it has been suggested that joint inflammation could be triggered by PAMPs common to various microorganisms interacting with cellular pattern-recognition receptors (PRRs). FLSs express a large number of PRRs, such as Toll-like receptor (TLR)-2, TLR-4, and TLR-9 [16], as well as numerous integrins. Their interaction with a variety of PAMPs may contribute to the aggressive phenotype of RA FLSs.

We reported previously that interaction of protein I/II, a cell wall component of oral streptococci, with FLSs triggers, through integrin α5β1, the production and release of inflammatory mediators such as IL-6 and IL-8 but not of TNF-α, IL-1, or IL-18 [17-19]. This cytokine synthesis involves extracellular signal-regulated kinase 1/2 and c-Jun N-terminal kinases, as well as activating protein-1-binding activity and nuclear translocation of nuclear factor κB [20]. Oral streptococci have been identified in the joints of RA patients [15] and have been shown to exacerbate collagen-induced arthritis in mice [21]. This study was undertaken to investigate whether a PAMP such as protein I/II could contribute to the aggressive behaviour of RA FLSs. Using cDNA array analysis, we show that this cell wall component triggers the synthesis of MMP-3 by FLSs and may therefore contribute to joint destruction. Furthermore, the genes modulated by protein I/II are mainly involved in cell signaling, protein turnover, and cellular communication, suggesting that protein I/II may contribute to the aggressive behavior of FLSs.

## Materials and methods

### Reagents

Cell-culture media (RPMI 1640 and M199), FCS, penicillin, streptomycin, amphotericin B, *Taq *DNA polymerase, dNTPs, and primers were from Invitrogen (Cergy-Pontoise, France). Cell-culture media never had an endotoxin level above 0.04 ng/mL, as tested by the *Limulus *chromogenic assay. LPS from *Escherichia coli *O55:B5, polymyxin B, and type XI collagenase were obtained from Sigma (Saint Quentin Fallavier, France). The First Strand cDNA synthesis kits and ^32^P dATP were from Amersham Pharmacia Biotech (Saclay, France). A Nucleospin RNA II extraction kit was from Macherey-Nagel (Souffelweyersheim, France). Atlas human cancer cDNA expression arrays were from Clontech (Ozyme, Saint Quentin Yvelines, France). BINDAZYME™ ProMMP-3 enzyme immunoassay kit was from The Binding Site (Saint Egreve, France). The SYBR^®^Green PCR master mix was from Applied Biosystems (Courtaboeuf, France). Buffers were prepared with apyrogenic water obtained from Braun Medical (Boulogne, France).

### Cell culture

Human FLSs were isolated from RA synovial tissues from three patients at the time of knee joint arthroscopic synovectomy, as described previously [22]. The diagnoses conformed to the revised criteria of the American College of Rheumatology [23]. Human FLSs were also isolated from osteoarthritis (OA) synovial tissues from three patients who were having joint replacements. FLS cultures were performed as previously described [20]. Briefly, tissues were minced, digested with 1 mg/mL collagenase in serum-free RPMI 1640 for 3 hours at 37°C, centrifuged (130 ***g ***for 10 minutes at 4°C) and resuspended in M199-RPMI 1640 (1:1) containing 2 mm l-glutamine, penicillin (100 IU/mL), streptomycin (100 μg/mL), amphotericin B (0.25 μg/mL), and 20% heat-inactivated FCS (complete medium). After overnight culture, nonadherent cells were removed and adherent cells were cultured in complete medium. At confluence, cells were trypsinized and passaged in 75-cm^2 ^culture flasks in complete medium containing 10% heat-inactivated FCS. Experiments were performed between the third and the ninth passages, during which time cultures were a homogeneous population of fibroblastic cells, negative for CD16 as determined by FACS analysis. Before activation experiments, cells were deprived of serum for 24 hours, and then the appropriate stimuli diluted in serum-free RPMI 1640 with antibiotics were added. To eliminate the possibility that the observed effects were due to LPS contamination, all the experiments were performed in the presence of polymyxin B (2 μg/ml). Cell numbers and cell viability were assessed using the MTT test (3-(4,5-dimethylthiazol-2-yl)-2,5-diphenyltetrazolium bromide test) as described elsewhere [24].

### Purification of protein I/II

Recombinant protein I/II of *Streptococcus mutans *OMZ 175 was purified from pHBsr-1 transformed *E. coli *cell extract by gel filtration and immunoaffinity chromatography as described elsewhere [25]. The purity of the protein was checked by SDS–PAGE after staining with Coomassie blue. Protein I/II migrated as a single band having an apparent molecular weight of 195 kDa.

### Stimulation of cells for total RNA extraction

FLSs (3 ×10^6 ^cells) were stimulated with 600 μL of serum-free RPMI 1640 containing protein I/II (125 pm final concentration). After a 4-hour incubation period, cells were centrifuged (130 ***g ***for 10 min at 4°C), and total RNA was extracted from cell pellets using the Nucleospin RNA II extraction kit in accordance with the manufacturer's instructions.

### Stimulation of cells for pro-MMP-3 assay

FLSs (5 ×10^3 ^cells) were grown to confluence in 96-well plates (7–10 days) and then stimulated with 200 μL of serum-free RPMI 1640 containing protein I/II (125 pm final concentration). After an 18-hour incubation period, a heterologous two-site sandwich ELISA was used to estimate pro-MMP-3 release in the culture supernatants.

### Gene expression profile analysis and quantification

The gene expression profile was examined using Atlas human cancer cDNA expression arrays, consisting of nylon membranes spotted in duplicate with cDNA fragments of 588 known genes. These genes are classified into several functional groups, such as oncogenes and tumor suppressors, growth factors and receptors, and regulators of cell adhesion, angiogenesis, and cell cycle (listed at ). Total RNA (2 μg) was converted into ^32^P-labeled first-strand cDNA following the protocol provided by the manufacturer. Briefly, total RNA was reverse-transcribed using MMLV (Moloney murine leukemia virus) reverse transcriptase and ^32^P-labeled dATP in a PCR thermal cycler set at 50°C for 25 min. Labeled cDNA probes were then purified from unincorporated ^32^P-labeled nucleotides and small (<0.1 kb) cDNA fragments using column chromatography. Probes (2–10 × 10^6 ^cpm) were freshly applied to cDNA array membranes to hybridize overnight at 68°C in continuously agitated roller bottles. After hybridization, membranes were washed four times, for 30 min each, at 68°C with 2 × SSC (standard saline citrate), 1% SDS, and once with 0.1 × SSC, 0.5% SDS, followed by one wash with 2 × SSC at room temperature. Array membranes were wrapped in plastic and exposed to a phosphor screen for 1–5 days, depending on the radiation intensity of the bound fragments. After image acquisition on a Storm phosphor imager (ImageQuant, Molecular Dynamics, Sunnyvale, CA, USA), spots were quantified using the AtlasImage 2.0 Software (Clontech), developed specifically for analysis of the Atlas cDNA expression arrays. All spots were individually checked by hand to ensure accuracy of the detection method. A dot was considered to be positive if it was well located and at least three times the local background level. Spots of poor quality were not included. For each patient, the relative expression level of each gene between protein-I/II-stimulated and control FLSs was evaluated and standardized based on expression levels of housekeeping genes included on each array.

### RT-PCR reactions

Total RNA (2 μg) isolated from FLSs was reverse-transcribed using the First Strand cDNA Synthesis Kit in accordance with the manufacturer's instructions. Total RNA (8 μL) was mixed with 5 μl of the bulk reaction mix, 1 μl of DTT (200 mm), and 1 μl of the Not I-d(T)_18 _bifunctional primer (0.2 μg/ml). The reaction was carried out for 1 hour at 37°C. Real-time PCR was performed in 96-well plates in a total volume of 25 μl using the SYBR^®^Green PCR master mix (containing SYBRGreen dye, AmpliTAq Gold^® ^DNA Polymerase, dNTPs with dUTP, passive reference and optimized buffer components) and gene-specific primers (250 nm): MMP-3 1) 5' GCA GTT TGC TCA GCC TAT CC 3' and 2) 5' GAG TGT CGG AGT CCA GCT TC 3' [26]; GAPDH (glyceraldehyde-3-phosphate dehydrogenase), 1) 5' AGC AAT GCC TCC TGC ACC ACC AAC 3' and 2) 5' CCG GAG GGG CCA TCC ACA GTC T 3' [27]. After incubation at 50°C for 10 min and at 95°C for 10 min, samples were subjected to 40 rounds of amplification for 15s at 95°C, 15s at 56°C, and 40s at 72°C using the AbiPrism 7700 Sequence Detection System (Applied Biosystems). Amplification products were detected as an increased fluorescent signal of SYBR^®^Green during the amplification cycles. Results were obtained using SDS Software (Applied Biosystems, Foster City, CA, USA) and evaluated using Excel (Microsoft). Primer efficiency was calculated for MMP-3 and GAPDH using the standard curve method in accordance with the supplier's recommendations. As the MMP-3 and GAPDH amplifications were about equally efficient, the relative expression levels of the MMP-3 gene were evaluated using the 2^-ΔΔCT ^method as described by Applied Biosystems. Additionally, to confirm the amplification specificity of each gene product, the PCR products were subjected to a melting-curve analysis.

### Pro-MMP-3 assay

Pro-MMP-3 levels in cell-culture supernatants were determined using the BINDAZYME™ pro-MMP-3 Enzyme Immunoassay Kit in accordance with the manufacturer's instructions. Briefly, duplicate samples were added to wells coated with anti-pro-MMP-3 and were then incubated with anti-pro-MMP-3 peroxidase conjugate; the peroxidase substrate (TMB) was then added. After the reaction had been stopped by the addition of phosphoric acid (3 m), the optical density of each well was read at 450 nm. Pro-MMP-3 levels were calculated using a standard curve.

### Statistical analysis

Values are represented as means ± standard error of the mean. The significance of the results was analyzed using Student's two-tailed *t*-test. Values of *P *< 0.05 were considered significant.

## Results

### Gene expression in RA and OA FLSs after a stimulation with protein I/II

**Figure 1:**
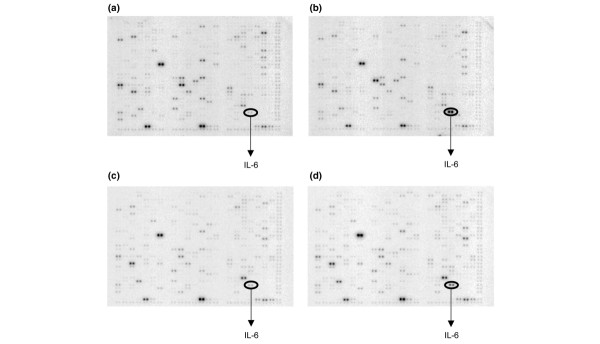
Gene expression patterns in rheumatoid arthritis (RA) and osteoarthritis (OA) fibroblast-like synoviocytes (FLSs). Total RNA of control RA **(a) **and OA FLSs **(c) **and of RA **(b) **and OA FLSs **(d) **stimulated for 4 hours with protein I/II were first reverse-transcribed and ^32^P-labeled and then hybridized to Atlas human cancer cDNA expression arrays. Arrows indicate double spots representing IL-6.

In this study we used a gene array technique to further investigate the cellular response of FLSs to protein I/II. As we were particularly interested in genes that could contribute to the aggressive behavior of RA FLSs, we chose Atlas human cancer cDNA expression arrays containing genes involved in cell-cycle regulation, cellular adhesion, and inflammation. In parallel to RA FLSs, we used OA FLSs in order to study the protein-I/II-induced response of FLSs isolated from a noninflammatory arthropathy. After a 4-hour incubation in the presence or absence of 125 pm protein I/II, the total RNA from FLSs of three RA and three OA patients was extracted, and radiolabeled cDNA probes were subsequently hybridized to the cDNA arrays. Figure 1 shows the expression patterns of FLSs isolated from one RA and one OA patient in nonstimulated controls after 4 hours (Fig. 1a,1c) and after stimulation with protein I/II for 4 hours (Fig. 1b,1d). The products of some differentially expressed genes (e.g. the gene for IL-6) are easily observed. The relative expression level of each gene in protein-I/II-stimulated FLSs versus control FLSs was evaluated with the AtlasImage 2.0 Software.

When a cut-off of a threefold change in mRNA expression was used, 28 genes were up-regulated and 5 genes were down-regulated in a least one of the three RA FLSs tested (Tables 1 and 2). These modulated genes account for about 6% of all profiled genes. Among the genes up-regulated by protein I/II in all three RA FLSs, two genes encoding proinflammatory cytokines, IL-6 and leukemia inhibitory factor, showed a strong expression level. The expression of other cytokine genes included on the array, such as TNF-α or IL-1, was not affected by protein I/II stimulation (IL-8 was not represented on the array). Protein I/II also strongly up-regulated the expression of MMP-3 (stromelysin 1), whereas the expression of genes encoding other members of the MMP family was not modified. Furthermore, a transcription factor, interferon regulatory factor 1 (IRF-1), as well as a growth factor, fibroblast growth factor-5 (FGF-5), were up-regulated by protein I/II in the three RA FLSs, although the expression levels were lower. Besides this group of genes which were strongly up-regulated in all RA FLSs, the expression of other genes was more heterogeneous and varied between the different RA FLS cultures. Among these genes, mainly involved in protein turnover and in cell signaling and communication, two genes, encoding CDC27Hs protein and interferon γ antagonist, were strongly up-regulated and the expression of three more genes (those encoding integrin αE, vimentin, and transmembrane protein SEX) was less intensely up-regulated in two of the three RA FLSs.

Interestingly, the expression of a few genes was down-regulated by protein I/II and only one gene was down-regulated in all three RA FLSs tested, namely, that for insulin-like growth factor binding protein-4. This member of the family of insulin-like growth factor binding proteins, which act as carriers and regulators of insulin-like growth factor, is believed to play an important role in maintaining the equilibrium between synthesis and degradation of tissue matrix molecules.

In OA FLSs, the number of genes modulated within 4 hours by protein I/II was much lower than in RA FLSs: protein I/II up-regulated the expression of only one gene, namely IL-6, in all OA FLSs tested. Other genes were up-regulated but with a high variability among FLSs cultures (Table 3). A very interesting finding was that one of the three OA FLS cultures (from patient 3) showed a gene expression pattern that was very close to the patterns obtained with protein-I/II-stimulated RA FLSs. Clinical investigations revealed that this patient had a secondary arthropathy of the knee occurring during a long remission phase of RA. This patient was therefore excluded from further experiments.

The up-regulation of IL-6 gene expression found in both RA and OA FLSs in response to protein I/II has been previously shown to be followed by an increase of IL-6 release from these cells (MB Zeisel and colleagues, unpublished data) [20]. Since we were primarily interested in genes that could contribute to the aggressive, invasive behavior of RA FLSs, the marked up-regulation of the MMP-3 gene, which plays a major role in cartilage degradation, prompted us to further study its expression in FLSs stimulated with protein I/II.

### Confirmation of the differential expression of MMP-3 by RT-PCR analysis

In order to confirm the up-regulation of the expression of MMP-3 gene in RA FLSs by protein I/II, we performed real-time PCR analysis for MMP-3. Total RNA was extracted from FLSs isolated from three RA FLSs stimulated with protein I/II or left untreated, reverse-transcribed, and then amplified by real-time PCR. MMP-3 mRNA showed a mean 36-fold up-regulation after protein I/II stimulation of RA FLSs in comparison with control cells, confirming the results obtained with the cDNA array experiments.

### Confirmation of the differential expression of MMP-3 by pro-MMP-3 assay

**Figure 2:**
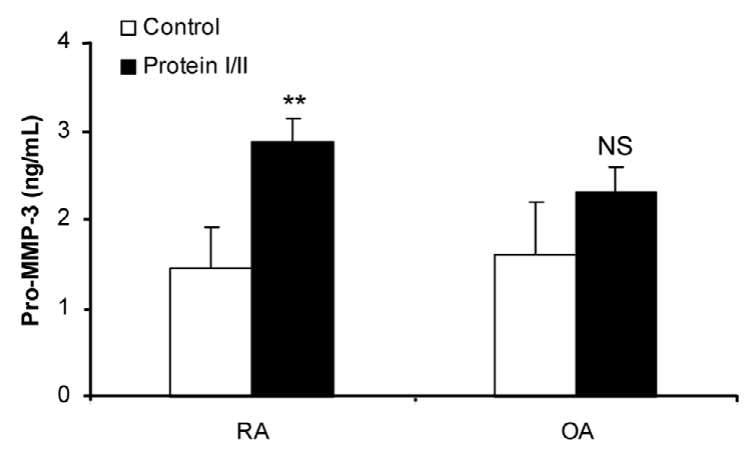
Protein-I/II-induced release of pro-matrix metalloproteinase (pro-MMP-3) from rheumatoid arthritis (RA) fibroblast-like synoviocytes (FLSs) and osteoarthritis (OA) FLSs. The level of pro-MMP-3 was determined in culture supernatants from control RA FLSs stimulated for 18 hours with protein I/II (*n *= 3) and OA FLSs (*n *= 3), using heterologous two-site sandwich ELISA. ELISA was performed in duplicate. Experiments were performed in duplicate. Values are expressed as means ± standard error of the mean. Statistical comparison of protein-I/II-stimulated and control samples was performed using the *t*-test (**P < 0.01; NS, not significant).

To determine whether the up-regulation of the expression of MMP-3 gene by protein I/II stimulation in RA FLSs resulted in changes in gene translation and thereby contributed to an increase in the release of pro-MMP-3, we next measured the level of pro-MMP-3 in culture supernatants from control and RA FLSs that had been stimulated with protein I/II for 18 hours. As MMP-3 is first synthesized as a proenzyme (pro-MMP-3), which is subsequently cleaved to generate active MMP-3, we chose to assay pro-MMP-3 levels, as the activation process might not occur in our *in vitro *cultures. Figure 2 shows that control RA FLSs basally released pro-MMP-3 (1.4 ± 0.4 ng/mL) and that stimulation with protein I/II induced an increase in pro-MMP-3 secretion (3.1 ± 0.4 ng/mL, *P *< 0.01). Control OA FLSs basally released pro-MMP-3, but protein I/II stimulation did not significantly increase pro-MMP-3 secretion from these cells (Fig. 2). To confirm the activation of FLSs, we assayed IL-6 and IL-8 levels in the same supernatants used for pro-MMP-3 assay, and found that protein I/II induced a significant increase in the release of these cytokines from both RA and OA FLSs (data not shown).

## Discussion

In this study, we analyzed gene expression changes in RA FLSs after stimulation with protein I/II, a ligand of integrin α5β1, a PRR highly expressed on FLSs. We chose to study the early induction of genes, within 4 hours of protein I/II stimulation, in order to analyze the direct effects of protein I/II, but we cannot rule out that some genes can be modulated in an autocrine fashion by some FLS-secreted mediators. Our results showed that stimulation of FLSs through this PAMP/PRR pathway induced the expression of several genes that may be involved in inflammation, matrix degradation, and invasiveness.

Protein I/II strongly up-regulated the expression of genes encoding IL-6 and leukemia inhibitory factor in RA FLSs. Although a great many interleukins (IL-1 to IL-17 except IL-8 and IL-16) as well as members of the TNF family were represented on the cDNA arrays, the expression of genes for only these two cytokines was modulated by protein I/II. IL-6 and leukemia inhibitory factor, which both belong to the same family of cytokines, are known to participate substantively in the pathogenesis of murine models of arthritis as well as RA. In fact, they contribute to cartilage degradation by inducing proteoglycan resorption and inhibiting proteoglycan synthesis [28].

Elevated expression of MMP-3 gene as well as pro-MMP-3 secretion in response to protein I/II were also found in RA FLSs, whereas the expression of other MMPs (MMP-1 to MMP-3 and MMP-7 to MMP-18) present on the arrays were not affected by protein I/II stimulation. FLSs have been reported to express different kinds of MMPs, but, as was found for cytokines, protein I/II seemed to up-regulate only a limited number of genes encoding matrix-degrading enzymes. Interestingly, the only MMP up-regulated by protein I/II is MMP-3, which is one of the major MMPs involved in cartilage destruction, and it has also been associated with an increased invasive potential of cells [29].

Kyburz and colleagues found similar cytokine and MMP expression profiles (i.e. IL-6, IL-8, and MMP-3) after stimulation of RA FLSs with the TLR-2 ligand peptidoglycan [16]. Peptidoglycan is one of the bacterial components that have been detected in the synovial cavity [9] and have been shown to induce an inflammatory response in the joint [13]. In contrast to our results, peptidoglycan not only induced the expression of MMP-3 but also seemed able to up-regulate the expression of other MMPs, such as MMP-1, MMP-9, and MMP-13 [16]. Furthermore, this bacterial component up-regulated the expression of several chemokine genes, including RANTES (regulated upon activation, normal T-cell expressed and secreted) and MCP-1 (monocyte chemoattractant protein-1) [30], but these genes were not represented on the arrays used in this study. These data indicate that various PRRs present on FLSs may contribute in different ways to the aggressive behavior of RA FLSs.

In this study, we also evaluated how FLSs isolated from OA patients responded to protein I/II. In our cDNA analysis, protein I/II induced strong IL-6 production in both RA and OA FLSs. In contrast to RA FLSs, OA FLSs did not express leukemia inhibitory factor or MMP-3. Our results are in contrast with those of Pierer and colleagues, who did not find a significant difference between RA and OA FLSs regarding MMP-3 and chemokine expression after stimulation with peptidoglycan. However, they found a higher chemokine level in the synovial fluid of RA patients than in OA patients [30]. The differences we noted between the gene expression profiles of protein-I/II-stimulated RA and OA FLSs could be due to particular features of RA FLSs but are probably not due to the absence of integrin α5β1, which is expressed on both RA and OA FLSs *in vitro *(data not shown).

Moreover, we have shown that protein I/II induced the expression of the transcription factor IRF-1, the growth factor FGF-5, and vimentin in RA but not OA FLSs. IRF-1 activates transcription from genes that play a role in inflammation, such as cyclooxygenase-2 and caspase 1, but also in apoptosis or cell growth inhibition [31]. In contrast to IRF-1, FGF-5 and vimentin are associated with increased cell growth, motility, and invasiveness [32,33]. Protein I/II seems thus able to act on FLSs apoptosis/proliferation by inducing different genes, although their effects may be opposite.

These PAMP–PRR interactions are probably not sufficient to explain activation of FLSs in RA and it is evident that additional factors, such as genetic polymorphisms, play an important role, because bacterial cell wall components, such as peptidoglycan, have also been found in joint tissues from OA patients but do not induce the inflammatory response occurring in RA. However, bacterial components probably have an important role in perpetuating joint inflammation. In support of this hypothesis, Choe and colleagues showed that innate immune functions via TLR-4 may perpetuate inflammatory mechanisms and bypass the need for IL-1 in chronic joint inflammation. In fact, in the transgenic K/BXN mice, IL-1 plays a key role in joint swelling and destruction, but administration of the TLR-4 ligand LPS along with arthritogenic serum from K/BXN mice resulted in joint swelling and destruction in IL-1R-deficient mice but not in MyD88-deficient mice [34].

Further experiments will be necessary to define the mechanisms underlying this PAMP-specific response of RA FLSs.

## Conclusion

Our results suggest that a bacterial ligand of integrin α5β1 may contribute to the aggressive behavior of RA FLSs by inducing the release of pro-inflammatory cytokines, such as IL-6, and a cartilage-degrading enzyme, MMP-3.

## Abbreviations

ELISA = enzyme-linked immunosorbent assay; FACS = fluorescence-activated cell sorter; FCS = fetal calf serum; FGF = fibroblast growth factor; FLS = fibroblast-like synoviocyte; GAPDH = glyceraldehyde-3-phosphate dehydrogenase; IL = interleukin; IRF = interferon regulatory factor; LPS = lipopolysaccharide; MMP = matrix metalloproteinase; OA = osteoarthritis; PAMP = pathogen-associated molecular pattern; PCR = polymerase chain reaction; PRR = pattern-recognition receptor; RA = rheumatoid arthritis; RT = reverse transcriptase; SSC = standard saline citrate; TLR = Toll-like receptor.

## Competing interests

The author(s) declare that they have no competing interests.

## Authors' contributions

MBZ carried out the study and drafted the manuscript. VAD participated in the study. DW and JS coordinated the study. All authors read and approved the manuscript.

## Figures and Tables

**Table 1:** Up-regulated genes in rheumatoid arthritis (RA) fibroblast-like synoviocytes (FLSs) stimulated with protein I/II. Confluent RA FLSs were stimulated with protein I/II for 4 hours or left untreated as controls. Total RNA was isolated, reverse-transcribed into ^32^P-labeled cDNA, and hybridized to Atlas human cancer cDNA expression arrays. The values are expression ratios (protein I/II/ control).

		RA patient		
		
Protein product of gene	GenBank accession no.	1	2	3
Matrix metalloproteinase 3	X05232	5.0	190.3	6.0
IL-6	X04602	14.6	65.0	4.0
Leukemia inhibitory factor	X13967	10.8	15.9	5.0
Interferon regulatory factor 1	X14454	9.4	15.3	3.4
Fibroblast growth factor-5	M37825	3.5	2.9	5.7
CDC27Hs protein	U00001	7.4	-	10.6
Interferon gamma antagonist	A25270	5.4	-	13.0
Integrin αE	L25851	3.9	-	3.5
Vimentin	X56134	3.2	-	3.7
Transmembrane protein SEX	X87852	3.8	-	3.0
TRK-T3	X85960	-	-	7.3
Collagen I α_2 _subunit	X55525	5.7	-	-
Transforming growth factor β2	M19154	-	-	4.8
Cell division protein kinase 4	M14505	4.7	-	-
Bone morphogenetic protein 2A	M22489	-	4.5	-
Rho-related GTP-binding protein RhoE	X95282	4.3	-	-
Cyclin H	U11791	4.3	-	-
Tyrosine-protein kinase receptor UFO	M76125	3.9	-	-
Tissue inhibitor of metalloproteinase 1	X03124	3.9	-	-
BIGH3	M77349	3.8	-	-
Ras-related C3 botulinum toxin substrate 1	M29870	3.6	-	-
Transforming protein RhoA H12	L25080	3.5	-	-
CD9 antigen	M38690	3.5	-	-
Notch homolog	M99437	3.4	-	-
Integrin β8	M73780	3.4	-	-
Secreted protein acidic and rich in cysteine	J03040	3.2	-	-
α_2_-Macroglobulin receptor	X13916	3.1	-	-
Fibronectin 1	X02761	3.0	-	-

-, not found or <3.0.

**Table 2:** Down-regulated genes in rheumatoid arthritis (RA) fibroblast-like synoviocytes (FLSs) stimulated with protein I/II. Confluent RA FLSs were stimulated with protein I/II for 4 hours or left untreated as controls. Total RNA was isolated, reverse-transcribed into ^32^P-labeled cDNA, and hybridized to Atlas human cancer cDNA expression arrays. The values are expression ratios (protein I/II / control).

		RA patient		
		
Protein product of gene	GenBank accession no.	1	2	3
Insulin-like growth factor binding protein-4	M62403	0.1	0.2	0.2
Insulin-like growth factor binding protein-5	M65062	-	0.2	-
Cyclin-dependent kinase 4 inhibitor D	U40343	-	-	0.3
Ubiquitin-conjugating enzyme E2A	M74524	-	-	0.3
Methallothionein III	D13365	0.3	-	-

**Table 3:** Up- and down-regulated genes in osteoarthritis (OA) fibroblast-like synoviocytes (FLSs) stimulated with protein I/II. Confluent OA FLSs were stimulated with protein I/II for 4 hours or left untreated as controls. Total RNA was isolated, reverse-transcribed into ^32^P-labeled cDNA, and hybridized to Atlas human cancer cDNA expression arrays. The values are expression ratios (protein I/II / control).

		OA patient		
		
Protein product of gene	GenBank accession no.	1	2	**3**^a^
*Up-regulated*				
IL-6	X04602	3.0	3.8	**6.8**
Interferon-gamma receptor β subunit	U05875	5.8	-	**7.7**
Interferon regulatory factor 1	X14454	-	11.0	**5.5**
Leukemia inhibitory factor	X13967	4.1	-	**5.0**
Matrix matalloproteinase 3	X05232	-	-	**13.3**
Placenta growth factors 1+2	X54936	-	-	**4.6**
Glia maturation factor β	M86492	-	-	**4.5**
Transforming growth factor β2	M19154	-	-	**4.5**
Growth inhibitory factor	D13365	-	-	**3.4**
Rho-related GTP-binding protein RhoE	X95282	-	-	**3.2**
Vascular endothelial growth factor receptor 1	X51602	-	-	**3.2**
Collagen VI α_3 _subunit	X52022	-	-	**3.2**
Early growth response protein 1	X52541	-	-	**3.0**
TRK-T3	X85960	-	-	**3.0**
*Down-regulated*				
Vimentin	X56134	-	0.3	**-**
Transmembrane protein SEX	X87852	-	0.3	**-**
Integrin β8	M73780	-	0.3	**-**

^a^Clinical investigations revealed that, besides OA, patient 3 (results in bold type) also had RA.
